# Rate and selectivity hysteresis during the carbon monoxide hydrogenation over promoted Co/MnOx catalysts

**DOI:** 10.1038/s41467-019-11836-z

**Published:** 2019-09-02

**Authors:** Yizhi Xiang, Libor Kovarik, Norbert Kruse

**Affiliations:** 1 Voiland School of Chemical Engineering and Bioengineering, Washington State University, Pullman, WA 99164 USA; 2 Environmental Molecular Science Laboratory, Pacific Northwest National Laboratory, P.O. Box 999, Richland, WA 99352 USA; 3 Institute for Integrated Catalysis, Pacific Northwest National Laboratory, Richland, WA 99332 USA; 4 Present Address: Dave C. Swalm School of Chemical Engineering, Mississippi State University, Mississippi State, MS 39762 USA

**Keywords:** Catalysis, Heterogeneous catalysis

## Abstract

While cobalt-based catalysts have been used in industrial Fischer-Tropsch synthesis for decades, little is known about how the dynamics of the Co-Co_2_C phase transformation drive their performance. Here we report on the occurrence of hysteresis effects in the Fischer-Tropsch reaction over potassium promoted Co/MnO_x_ catalyst. Both the reaction rate and the selectivity to chain-lengthened paraffins and terminally functionalized products (aldehydes, alcohols, olefins) show bistability when varying the hydrogen/carbon monoxide partial pressures back and forth from overall reducing to carbidizing conditions. While the carbon monoxide conversion and the selectivity to functionalized products follow clockwise hysteresis, the selectivity to paraffins shows counter-clockwise behavior. In situ X-ray diffraction demonstrates the activity/selectivity bistability to be driven by a Co-Co_2_C phase transformation. The conclusions are supported by High Resolution Transmission Electron Microscopy which identifies the Co-Co_2_C transformation, Mn_5_O_8_ layered topologies at low H_2_/CO partial pressure ratios, and MnO at high such ratios.

## Abstract (web summary)

The question for the structure-reactivity relationship of cobalt-based catalysts for the carbon monoxide hydrogenation is as old as the reaction itself. Here the authors show kinetic hysteresis for a potassium-promoted cobalt-manganese oxide catalyst to be driven by the Co-Co_2_C phase transition.

## Introduction

Catalytic reactions occur far from equilibrium. Consequently, multiple steady states may be observed along with kinetic hysteresis effects. Both clockwise and counter-clockwise dynamic hysteresis may occur upon variation of critical reaction parameters. The reason for the occurrence of hysteretic response behavior can frequently be related to competitive and asymmetric reactant adsorption or, more generally, to inhibition effects following, for example, concentration-dependent catalyst phase transitions. While considerable foundational knowledge has been acquired over the past three decades on kinetic non-linearities of seemingly simple catalytic surface reactions like the CO oxidation or NO_x_ reduction^1^, much less information is presently available for complex reaction networks with varying product selectivity. An excellent example is the CO hydrogenation according to Fischer–Tropsch (F–T), which may lead to the production of chain-lengthened hydrocarbons and terminally functionalized oxygenates over Co-based and Fe-based catalysts^2,3^.

Historically, one of the earliest observations of a F–T reaction-induced chemical phase transition was made for Fe-based catalysts^4^, which were found to transform into carbide structures through pre-carbidization of iron oxide with CO or syngas^5^ or, more recently, during the ongoing F–T reaction using Fe(0)^6–8^ as activated precursor. By contrast, bulk cobalt carbides were commonly not observed, and therefore not considered as being relevant for the construction of the catalytically active phase^9–16^. Moreover, bulk cobalt carbides were actually considered thermodynamically metastable since they decomposed under inert atmosphere and in the presence of H_2_ at elevated temperatures^17,18^. Mohandas et al.^15^ and Claeys et al.^19^ observed that, under F–T reaction conditions, as-prepared Co_2_C can be converted into metallic cobalt at temperatures above 220 °C. Nevertheless, the formation of Co_2_C was occasionally observed through exposure of reduced cobalt to pure CO and found to be strongly dependent on the choice of the support material and time-on-stream in syngas^16,20^. More recently, Paterson et al. reported a nice demonstration of Ostwald’s Stufenregel (“rule of stages”) when transforming Co_3_O_4_ spinel stepwise into hexagonal Co_2_C by exposure to CO^21^. Particular attention received on Co_2_C formation during F–T synthesis over Co-based catalysts promoted by Mn and/or alkali^22–27^. Such catalysts demonstrated a remarkable capacity in producing terminally functionalized products, such as n-aldehydes^22^, n-alcohols^25,28,29^, or olefins^23,24,26,27^. The promoting effect of Mn on the formation of Co_2_C during the F–T reaction was recently investigated by Ding et al.^25^. The authors suggested that Mn facilitates the dissociation and disproportionation of CO and suppresses H_2_ adsorption on the catalyst surface, thereby creating a relatively C-rich and H-lean surface chemical environment. Similar arguments were previously advanced by Johnson et al.^30,31^ to explain the high selectivity of C_5+_ products and olefins, at the expense of methane. Overall, however, little is known about how the kinetics of the Co–Co_2_C phase transformation drive both reaction rate and selectivity.

Here, we show kinetic hysteresis in the F–T reaction and identify compositional changes of the catalyst to drive this hysteresis in the absence of thermokinetic effects. Using a potassium-promoted Co/MnO_x_ catalyst with Co_4_Mn_1_K_0_._1_ metal atomic composition, which was previously shown to produce more than 60 wt% n-aldehydes under H_2_-lean conditions of the F–T reaction^22^, we will demonstrate the occurrence of rate and selectivity hysteresis while cycling the syngas composition from high P_H2_/P_CO_ to low and back to high such ratio. Two steady states with distinct rate-selectivity signatures are produced this way for a unique point in the parameter space; only the direction of the P_H2_/P_CO_ variation matters when approaching such points within the bistability regime. This history dependence of the system will be shown to be exclusively dependent on and driven by the kinetic Co–Co_2_C phase transition.

## Results

### Catalytic results

**Fig. 1:**
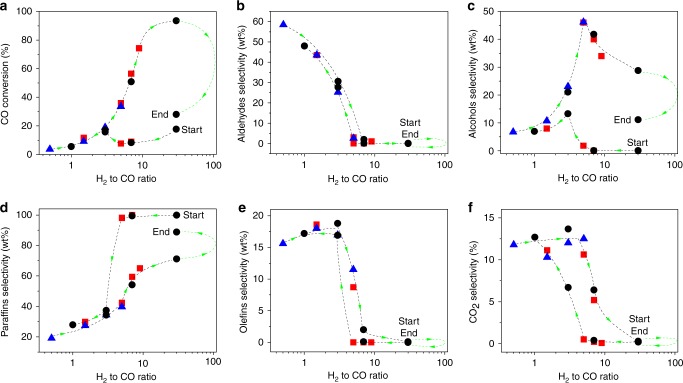
Kinetic hysteresis in the catalytic CO hydrogenation. **a** CO conversion; **b**–**e** selectivities of aldehydes, alcohols, paraffins and olefins, respectively (ex CO_2_); **f** CO_2_ selectivity. The same batch of catalyst is tested independently three times (circle), (square), and (triangle) over 0.5 g Co_4_Mn_1_K_0_._1_ at 220 °C, 40 bar, and GHSV = 3600 h^−1^. During each run, the P_H2_/P_CO_ ratio is varied systematically as follows: (circle) 30/1 → 7/1 → 3/1 → 1/1 → 7/1 → 30/1 → 30/1 (after H_2_ treatment for 8 h: green dashed line), (square) 7/1 → 5/1 → 1.5/1 → 5/1 → 7/1 → 9/1, (triangle) 0.5/1 → 1.5/1 → 3/1 → 5/1. Each data point reflects quasi-steady-state conditions after 12 h time-on-stream

We first turn to our catalytic results, which are shown in Fig. 1. Obviously, both the CO conversion and the selectivity depend on whether the P_H2_/P_CO_ ratio is increased or decreased. Each data point in Fig. 1 represents quasi-steady-state behavior after ~12 h time-on-stream. We also note that three samples of the same batch of Co_4_Mn_1_K_0_._1_ catalyst are being investigated while varying the P_H2_/P_CO_ ratio. The results of these measurements fit into the same Fig. 1 without difficulties. With regard to the catalytic activity, the CO conversion (panel a) obviously shows clockwise hysteresis, i.e., a low reactivity state (LRS) is followed when decreasing the P_H2_/P_CO_ ratio from initially high (30/1) to low and, vice versa, a high-reactivity state (HRS) is followed when increasing this ratio back to high again. In particular, while the CO conversion is up to 20%, at the most (at P_H2_/P_CO_ = 30), and less than 10% for 5 ≤ P_H2_/P_CO_ ≤ 10, on the LRS branch, it increases significantly to 75% at P_H2_/P_CO _= 9 and up to 90% at P_H2_/P_CO _= 30 on the HRS branch. The bifurcation between LRS and HRS seems to occur at P_H2_/P_CO_ ~3. The hysteresis loop finally closes by running the catalyst under pure H_2_, i.e., removing CO from the reaction mixture at 220 °C and 40 bar (indicated by a green dashed line in Fig. 1). A similar hysteresis distinguishing the low reactivity from the high-reactivity branch is obtained when plotting the specific reaction rate rather than conversion (Supplementary Fig. 1). The slight misfit between “start” and “end” in both conversion and rate hysteresis (as well as the selectivity hysteresis discussed below) is probably associated with irreversible changes of the Co metallic phase which is mainly fcc besides less hcp when starting and rather equally distributed between hcp and fcc when finishing the experiments (see below for XRD and HRTEM results). We note that the transition from H_2_/CO = 30 to pure H_2_ (rather than systematically establishing higher such ratios) has technical reasons related to the back pressure control at very low CO gas pressures.

While CO conversion follows clockwise hysteresis, both clockwise and counter-clockwise hysteresis are observed for selectivity. Most notable hysteresis loops occur for paraffins and alcohols. Starting with essentially 100% selectivity (of which 70% are allotted to methane, see Supplementary Fig. 2a for methane selectivity hysteresis, at P_H2_/P_CO_ = 30/1), the yield in paraffins drops significantly at P_H2_/P_CO_ ≤ 5/1. Under partial pressure conditions typically applied in F–T synthesis (1 ≤ P_H2_/P_CO_ ≤ 3), paraffin selectivities are between 30 and ~40%. For under-stoichiometric reaction conditions, P_H2_/P_CO_ = 0.5, only 20% of paraffins are detected. When increasing the P_H2_/P_CO_ ratios from low to high, paraffin selectivities set out on their way back, but remain considerably lower than those of the forward run. A (nearly) closed hysteresis loop is obtained when subjecting the catalyst to pure H_2_ conditions. Thus, compared with the clockwise hysteresis of the CO conversion, an overall counter-clockwise behavior is seen for the paraffin selectivity. Interestingly, terminal alcohols and total oxygenates (see Supplementary Fig. 2b for total oxygenates selectivity hysteresis) run counter paraffins, i.e., show clockwise hysteresis: while alcohol selectivities are low for initially high P_H2_/P_CO_ ratios, they are significantly higher when increasing these ratios from low to high. A maximum alcohol production of ~45% is found for P_H2_/P_CO_ = 5. Larger such ratios cause a gradual decline of alcohols.

Turning to aldehydes and olefins, we note that both products can be easily hydrogenated into alcohols and paraffins, respectively, under hydrogen-rich conditions. Therefore, the hysteresis loops for aldehydes and olefins are much less distinct and absent for P_H2_/P_CO_ ≥ 5. Remarkably, while neither aldehydes nor olefins are ever produced for P_H2_/P_CO_ > 5, overall 60% of aldehydes and 15% of olefins (ex CO_2_) are obtained at P_H2_/P_CO_ = 0.5. Under these conditions, methane formation is low (~5%), see Supplementary Fig. 2a. More detailed product distributions along the LRS and HRS branches under the same reaction conditions can be found in Supplementary Fig. 3.

Finally, CO_2_ formation also shows significant clockwise hysteresis. For very high partial pressure ratios, P_H2_/P_CO_ > 9, no CO_2_ at all is formed. The highest CO_2_ selectivites occur at low such ratios, but remain below ~13% all the time. The observed hysteresis suggests CO_2_ formation to involve similar surface precursors as hydrocarbons and oxygenates rather than originating from the water–gas shift reaction, CO + H_2_O ↔ CO_2_ + H_2_.

Hysteresis effects as reported in this study raise the question for the intrinsic kinetics by which the steady states of the reaction are approached. We therefore measured the time dependence of changes in activity and product selectivity after switching the partial pressure ratios. One such measurement, carried out for a switch in P_H2_/P_CO_ from 5/1 to 1.5/1, is provided in Supplementary Fig. 4. The data clearly show a nonlinear response behavior consisting in moderate changes of the product selectivity during the first 5 h time-on-stream (TOS) followed by a rapid drop of paraffins and raise of olefins (from < 5 to 40%) and oxygenates (from < 5 to 20%) within a short period of time. For 6 ≤ TOS ≤ 12, oxygenates further increase to reach a selectivity of ~50% at the expense of both olefins and paraffins; close-to-steady state behavior for all product classes is obtained after ~12 h.

**Fig. 2:**
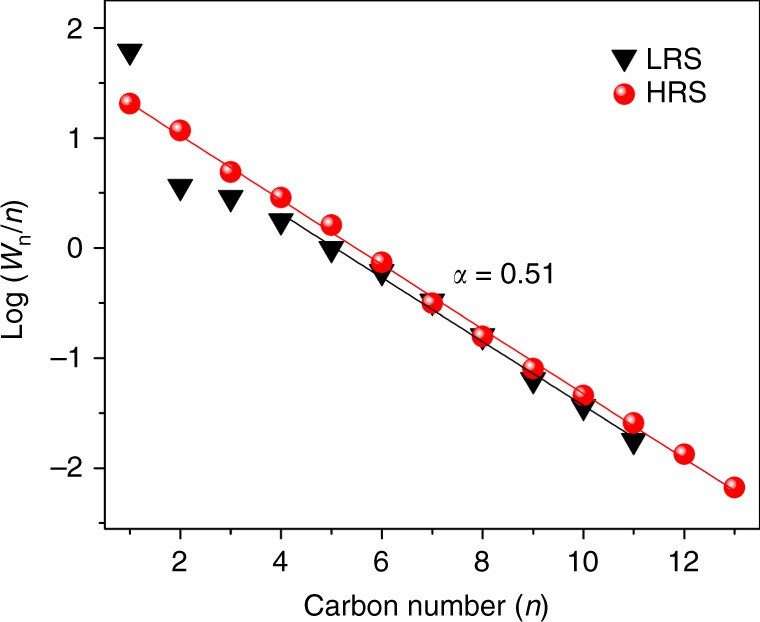
ASF distribution for low and high-reactivity hysteresis branches. Obtained at a H_2_/CO partial pressure ratio of 5/1 and otherwise identical reaction conditions as in Fig. 1. W_*n*_ stands for the mass fraction of all products containing n carbon atoms: W_*n*_ = W_*n*, paraffins_ + W_*n*, olefins_ + W_*n*, alcohols_ + W_*n*, aldehydes_

Product distributions in Fig. 1 have also been evaluated in terms of chain lengthening probabilities. Anderson–Schulz–Flory (ASF) plots have been constructed and are compiled in Fig. 2. Accordingly, a linear ASF behavior with unique chain lengthening probability α = 0.5 is obtained for C_4+_ product distributions on both the LRS and HRS branch. However, significant deviations from the linear ASF behavior are seen for C_1_–C_3_ on the low rate branch. These deviations seem to be general; they also occur at pressure ratios other than P_H2_/P_CO_ = 5 and are probably associated with variations in the composition of the “most abundant surface (reaction) intermediate (“masi” or “mari”).

### Structural characterization

**Fig. 3:**
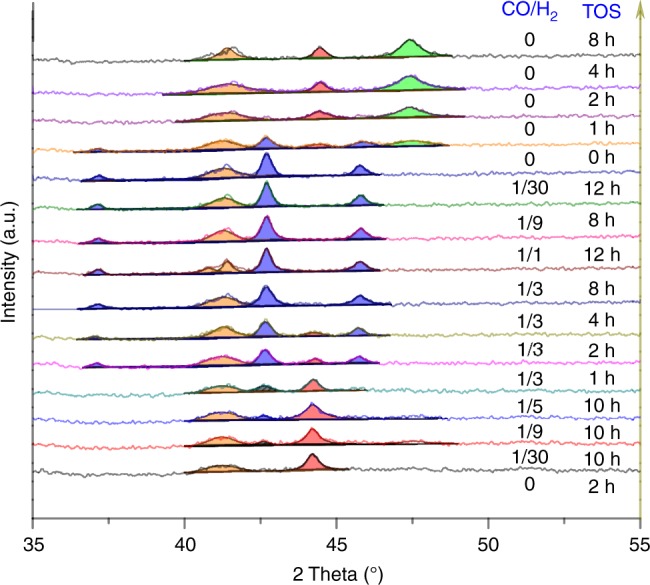
In situ XRD of a Co_4_Mn_1_K_0_._1_ catalyst. The results refer to a reaction temperature of 220 °C and atmospheric pressure. The same batch of catalyst was sequentially (from low to up) exposed to pure H_2_ or syngas (at various p_CO_/p_H2_) for different times-on-stream (TOS), see also Fig. S5. Red peaks indicate fcc cobalt (111), blue and green peaks are due to Co_2_C and hcp cobalt, respectively. Orange peaks at 2θ~41.3° are either Co_2_C (020) or hcp Co

To demonstrate that changes in the structure and composition of the catalyst correlate with the occurrence of kinetic hysteresis effects as described above, we have performed X-ray diffraction (XRD) experiments. To ensure this correlation also applies under in-operando (atmospheric) conditions, some of the XRD experiments have been made while following the catalytic production on-line. The general trend of hysteretic reaction behaviors has been reproduced this way. In Fig. 3, we focus on in situ XRD experiments to systematically follow the bulk Co composition for varying P_H2_/P_CO_ ratios. As seen in this Fig. (and also in Supplementary Fig. 5), only pure metallic cobalt phases (mainly represented by the (111) plane of Co fcc at 2θ ~44.3^°^, JCPDS 15–0806) are observed for virgin Co_4_Mn_1_K_0_._1_ after treatment in pure H_2_. Almost identical diffraction patterns occur after exposing the catalyst to H_2_-rich syngas (P_H2_/P_CO_ = 30/1) for 10 h. While metal diffraction remains the main phase when increasing the CO partial pressure to adjust P_H2_/P_CO_ = 9/1 and 5/1, trace amounts of Co_2_C diffraction are observed at 2θ ~42.5^°^ (JCPDS 05–0704). Interestingly, metallic cobalt diffraction fades away when further increasing the CO partial pressure to reach P_H2_/P_CO_ = 3/1; after 8 h time-on-stream at this pressure ratio hardly any metallic Co diffraction persists (for a detailed evolution of cobalt and Co_2_C diffraction lines as a function of time-on-stream see Supplementary Fig. 5). The diffraction lines (blue) centered at 2θ of 37.1, 41.4, 42.5, and 45.8^°^ correspond to the (011), (020), (111), and (210) planes of Co_2_C (JCPDS 05–0704). For a partial pressure ratio P_H2_/P_CO_ = 1/1, Co_2_C is definitely the only diffraction phase left. Strikingly, when decreasing the CO partial pressure from high back to low again (P_H2_/P_CO_ = 9/1 and 30/1) no change in the diffraction pattern is encountered. We therefore conclude that the back transformation of the Co_2_C phase to metallic Co is slow under H_2_-rich syngas conditions. However, after exposing the catalyst to pure H_2_ at 220 °C for 2 h, both fcc and hcp cobalt phases are retrieved. This behavior is reminiscent of reports on the Co_2_C metastability in the presence of H_2_^18^ and the decomposition of Co_2_C to hcp cobalt and graphite^32^. On the other hand, cobalt carbide formation under H_2_-rich (H_2_/CO = 9) F–T synthesis conditions was reported by Ducreux et al.^33^. Different from these earlier reports, our measurements clearly establish a hysteresis effect in the Co–Co_2_C reversible transition. Closed hysteresis loops involving the complete back transformation of Co_2_C into metallic Co are obtained after treatment in pure H_2_ (or at very high P_H2_/P_CO_ ratios >30).

**Fig. 4:**
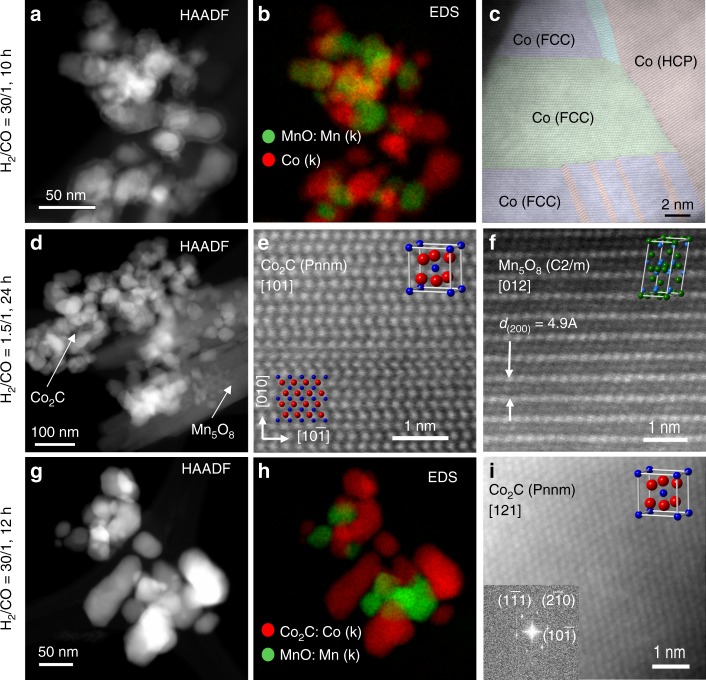
HRTEM images along with EDS chemical mapping. The TEM image of the Co_4_Mn_1_K_0_._1_ catalyst was obtained after CO hydrogenation at 220 °C, P_total_ = 40 bar. The partial pressure ratio is cycled between **a**–**c**: H_2_/CO = 30, **d**–**f**: H_2_/CO = 1.5, and **g**–**i**: back to H_2_/CO = 30. Sample history and time-on-stream (TOS): **a**–**c** fresh catalyst after 12 h TOS, **d**–**f**: fresh catalyst after 24 h TOS, **g**–**i**: catalyst subjected to H_2_/CO = 1.5 for 12 h first (as in **d**–**f**), then to successively increasing H_2_/CO ratios up to 30 (for another 12 h TOS). Scale bar for HAADF 50 nm, scale bar for HRTEM 1 nm

The correlation of a reversible chemical reconstruction of Co particles and the catalytic performance in terms of activity and selectivity ultimately lead to a remarkable hysteresis behavior, in which Co metallic phases correlate with high paraffin and Co_2_C with high oxygenate/olefin selectivity. However, questions as to the influence of the MnO_x_ phase as a dispersant and/or promoter persist and will be tackled by high-resolution transmission electron microscopy (HRTEM), along with energy-dispersive X-ray spectroscopy (EDS). To allow an as close as possible correlation of the catalytic test procedures preceding HRTEM/EDS measurements with those leading to kinetic hysteresis effects as shown in Fig. 1, we have investigated the same batch of Co_4_Mn_1_K_0_._1_ catalyst after reaction with P_H2_/P_CO_ of 30 (Fig. 4a–c) and 1.5 (panels (d–f) of the same Fig.) for 12 h using a fresh charge of catalyst in both cases. To mimic possible “memory” effects in the chemical composition of the catalyst while changing the P_H2_/P_CO_ ratio from low to high, we have subjected another sample charge to low such ratios (P_H2_/P_CO_ = 1.5) before adjusting the end-of-the-loop value P_H2_/P_CO_ = 30. As can be seen in Fig. 4, both metallic and oxidic phases undergo significant structural and chemical alterations while varying the P_H2_/P_CO_ ratio. The upper panel demonstrates Co metal particles to be mainly present in fcc and (to somewhat lesser extent) hcp phases under hydrogen-rich reaction conditions. It is interesting to note that bulk XRD has allowed identifying fcc Co rather than hcp, see Fig. 2. Running the catalytic reaction under CO-rich conditions, P_H2_/P_CO_ = 1.5 (Fig. 4d–f), causes metallic Co to entirely transform into Co_2_C and MnO_x_ to develop lath-like Mn_5_O_8_ (Mn(II)_2_Mn(IV)_3_O_8_). The occurrence of the latter is in agreement with our previous studies under similar reaction conditions^22^. Most surprisingly, when moving from low P_H2_/P_CO_ ratios back to higher ones (Fig. 4g–i), major amounts of Co_2_C persist these strongly reducing conditions while Mn_5_O_8_ decomposes to develop smaller MnO aggregates with cubic rock-salt structure. Metallic Co phases reappear very slowly in mainly hcp and lesser fcc crystal structure at P_H2_/P_CO_ = 30.

## Discussion

We have shown in this communication that prominent structural changes of a potassium-promoted Co/MnO_x_ catalyst occur during the Fischer–Tropsch reaction. The systematic variation of the H_2_/CO partial pressure ratios has allowed us to follow the reversible Co–Co_2_C bulk phase transformation as a function of time using in situ XRD, complemented by representative ex situ HRTEM studies. Once being formed at low such ratios, Co_2_C has proven to be rather perseverant with regard to its reduction to metallic Co. This observation, which is not in accordance with earlier reports claiming the Co_2_C phase to be “metastable”^17,18^, seems to be a key factor responsible for the activity–selectivity hysteresis of the CO hydrogenation. Ignoring in a first approximation the influence of the MnO_x_ phase and that of the potassium promoter, it seems that the occurrence of Co_2_C provides a positive feedback on chain-lengthened oxygenates and olefins formation. It is remarkable that at stoichiometric (P_H2_/P_CO_ = 2/1) as well as under-stoichiometric reaction conditions the combined selectivities of functionalized hydrocarbons range between ~70 and ~83%, at rather low CO conversion, while under H_2_-rich conditions, after entering the high-reactivity hysteresis branch, the product spectrum simplifies to ~35% 1-alcohols and 65% straight paraffins at a CO conversion of nearly 75% (P_H2_/P_CO_ = 9/1).

In a previous study with Mn(II)-promoted Co/SiO_2_, Johnson et al.^30,31^ considered the high selectivity of C_5+_ formation with their catalyst as being due to an increase of CO_ad_ concentrations, i.e., a decrease of the H_ad_/CO_ad_ ratios, following the strong Lewis acid–base interaction between Mn(II) and CO_ad_. While the present paper provides clear evidence for the partial pressure ratio P_H2_/P_CO_ to have a profound influence on the selectivity of the reaction, the amounts of Mn(II) (in MnO) or Mn(II)–Mn(IV) (in Mn_5_O_8_) employed in our study are far beyond the low Mn(II) promoter concentrations in Co/SiO_2_. To the best of our knowledge, neither Co/SiO_2_ nor Co/Al_2_O_3_ catalysts have ever been reported to produce significant amounts of chain-lengthened oxygenates during CO hydrogenation. Such catalysts have also not been observed to undergo reaction-induced structural changes involving a Co–Co_2_C bulk phase transition. We therefore conclude, in agreement with others^23–27^, that this phase transition is MnO_x_ mediated. Different from Si(IV) or Al(III), the valence fluctionality between Mn(II) and Mn(IV) seems to play an important role in the bulk transformation of Co into Co_2_C and is therefore key to understanding the rich variety of functionalized products in the CO hydrogenation over CoMn-based catalysts.

The significant activity–selectivity hysteresis reported in this communication may raise the question for the possible occurrence of oscillatory behavior. So far, we have not yet observed any. If such periodic change in activity and selectivity occurs for the present system, under isothermal conditions and in the absence of mass transport limitations, we would anticipate a rather long-period behavior due to the slowness of the Co–Co_2_C phase transition (speculating that this process would provide a suitable feedback for autonomous oscillations). Long-period oscillations are not unusual^34^ and were, for example, detected for the NO/hydrogen reaction over Rh single-crystal surfaces^35^. In this case, the reversible oxidation of the Rh surface region was identified to provide the necessary feedback. A recent paper by Suchorski et al.^36^ reported intriguing results on the occurrence of multifrequential oscillations in the O_2_–H_2_ reaction over Rh and demonstrated the importance of reversible subsurface oxygen diffusion.

We finally note that nonisothermal self-sustained rate oscillations in the F–T reaction were previously reported by Tsotsis et al.^37^. Both the reactor design as well as the catalyst system (Fe/ZSM-5) were very different from ours, though. Due to the exothermicity of the reaction, the authors observed peak-to-peak amplitudes of about 150 °C in the reactor temperature. Such large jumps in temperature are clearly absent under our experimental conditions. The discovery of rate-selectivity hysteresis, as reported in this paper, opens new avenues to tune the catalytic performance of the Co-based Fischer–Tropsch reaction. We anticipate bistability not to be limited to potassium-promoted Co/MnO_x_ catalysts but to apply to other systems with different selectivity signatures as well.

## Methods

### Catalyst preparation

Co_4_Mn_1_K_0.1_ catalyst was prepared via oxalate co-precipitation followed by thermal activation. To prepare the Co–Mn–-K mixed oxalate precursor, first a solution of both Co(NO_3_)_2_.6H_2_O and Mn(NO_3_)_2_.4H_2_O in acetone (100 ml), an aqueous solution of KNO_3_ (5 ml), and an acetone solution of H_2_C_2_O_4_. 2H_2_O (150 ml) were prepared in three separated beakers. Then the mixed acetone solution of Co(NO_3_)_2_.6H_2_O and Mn(NO_3_)_2_.4H_2_O together with the aqueous solution of KNO_3_ were added fast and simultaneously, under vigorous stirring, to the solution of H_2_C_2_O_4_. 2H_2_O. Stirring was kept for at least 5 min until the color of the precipitates appeared homogeneous. Then the slurries were kept overnight for aging. After removal of the supernatant acetone, the precipitate was centrifuged and dried overnight at 110 °C. The obtained oxalate precursor was then treated thermally in the presence of hydrogen through stripping of CO_2_ under H_2_ (30 ml min^−1^) at 370 °C for 1 h. After activation, the BET surface area of the catalyst was ~50 m^2^/g.

### Catalyst characterization

X-ray diffraction (XRD) patterns of the catalysts were collected with a Cu Kα source using a Rigaku Miniflex-600 X-ray diffractometer operating at 40 mA and 35 kV in the continuous-scan mode with steps of 1 degree/min in a wide 2θ angle range from 20 to 80°.High resolution Transmission electron microscopy (TEM) studies were performed with aberration corrected FEI Titan 80-300 operated at 300 kV. The instrument is equipped with a CEOS GmbH double-hexapole aberration corrector for the probe forming lens, which allows angstrom level resolution in scanning imaging modes. The present observations were performed in scanning mode using an HAADF detector. The probe convergence angle was 18 mrad, with an inner collection angle of 52 mrad. The EDS maps were collected with an aberration corrected Scanning Transmission Electron Microscope (STEM) (JEOL-ARM200F) operated at 200 kV. The instrument is equipped with a 100mm^2^ Silicon Drift Detector (~0.7srad, JEOL Centurio), allowing for high efficiency Energy Dispersive X-ray Spectroscopy. Acquisition and evaluation of the spectra was performed by NSS Thermo Scientific software package. In general, the STEM sample preparation involved mounting powder samples on copper grids covered with lacey carbon support films, and then immediately loading them into the TEM airlock.

### Catalytic testing

High-pressure catalytic tests were performed in a fixed-bed plug-flow reactor (Φ_inner_ = 7 mm). Typically, 1.3 g of oxalate were diluted with up to 2 g of SiC to achieve isothermal plug-flow conditions followed by in situ TPDec in H_2_ at 0.1 MPa (30 ml min^−1^) and 370 °C for 1 h (after oxalate decomposition, the amount of activated catalyst is around 0.5 g). The reactor was subsequently cooled to temperatures below 100 °C in flowing hydrogen before adding CO to produce a syngas feed with a specific H_2_/CO pressure ratio. Metal carbonyls (mainly Ni(CO)_4_) were removed by passing the CO feed through a zeolite 4A trap at high temperature before introduction into the reactor. The total flow rates (H_2_ + CO) were fixed at 40 ml min^−1^ providing GHSV = 3600 h^−1^ (Gas Hourly Space Velocity). After pressurizing the system to 40 bar, the temperature for the catalytic tests was raised using low heating rates of 1 °C min^−1^ to 220 °C. Catalytic activities and product selectivities were determined after stabilization for at least 12 h, and measured by an online GC-MS (*Agilent 7890A GC/5975 MS*).

## Supplementary information

Supplementary Information

Peer Review File

## Data Availability

Any data that support the plots and calculations within this paper as well as other findings of this study can be available from the corresponding author upon reasonable request.
