# Supplementary material for: Rate and selectivity hysteresis during the carbon monoxide hydrogenation over promoted Co/MnOx catalysts
Source: Nat Commun. 2019 Sep 2;10:3953. doi: 10.1038/s41467-019-11836-z (PMC6718517; doi:10.1038/s41467-019-11836-z)
Supplement: Supplementary file 1 — Supplementary Information [file 41467_2019_11836_MOESM1_ESM.pdf]

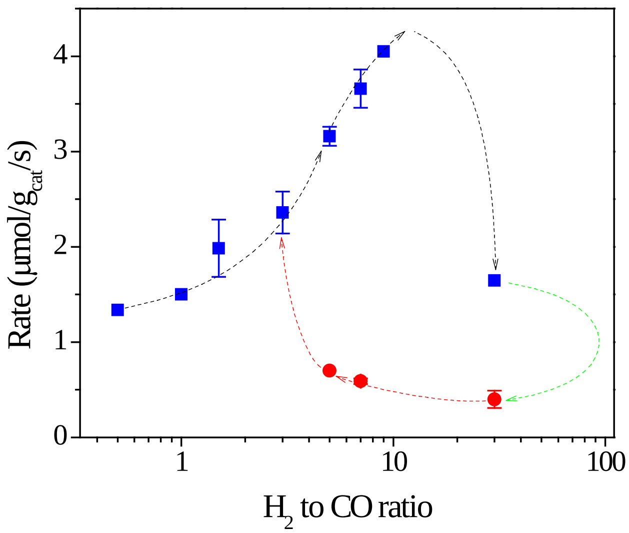

Supplementary Figure 1. Specific rate hysteresis in CO hydrogenation over $Co_4Mn_1K_{0.1}$ catalyst. Reaction conditions: 220°C, 40 bar and GHSV=3600 h^{-1}. The same catalyst has been tested independently for three times. During each run, the same batch of catalyst has been tested consecutively at different p_{H2}/p_{CO} conditions as shown in Figure 1. Reaction rates from the three runs were plotted in the same figure, error bars were obtained from the deviations of three individual measurements.

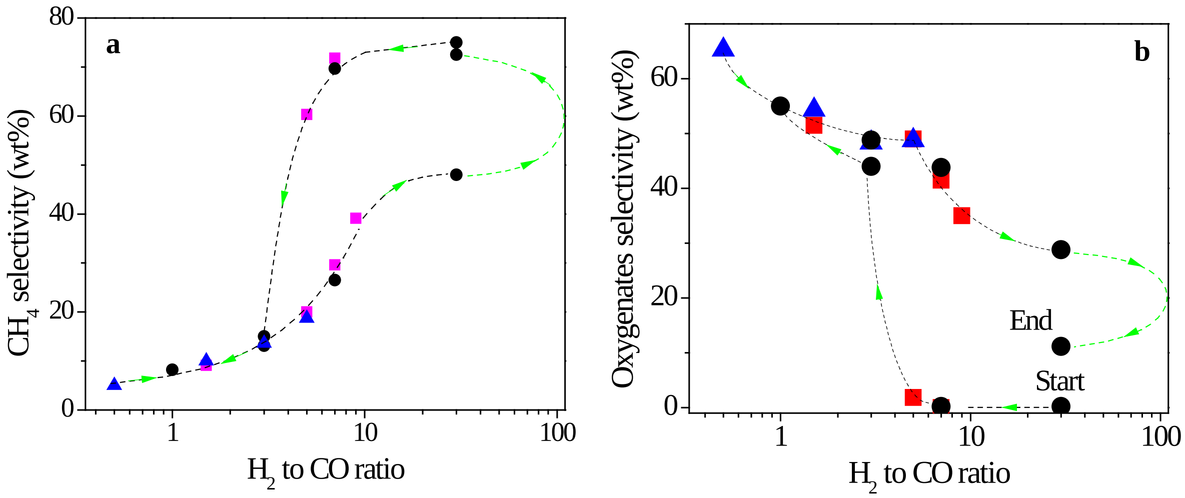

Supplementary Figure 2. Kinetic hysteresis with $Co_4Mn_1K_{0.1}$ catalyst in CO hydrogenation. (**a**) CH_4 selectivity and (**b**) total oxygenates selectivity. Reaction conditions: 220°C, 40 bar and GHSV=3600 h^{-1}. The same catalyst has been tested independently for three times (circle), (square) and (triangle). During each run, the same batch of catalyst has been tested consecutively at different P_{H2}/P_{CO} conditions: (circle) 30/1→7/1→3/1→1/1→7/1→30/1→30/1 (after H_2 treatment for 8 h: green dash line), (square) 7/1→5/1→1.5/1→5/1→7/1→9/1, (triangle) 0.5/1→1.5/1→3/1→5/1. While the oxygenates selectivity follows clockwise hysteresis, counter-clockwise hysteresis is observed for methane selectivity. Oxygenates selectivity is negligible for P_{H2}/P_{CO} between 30/1 and 5/1 after starting the experiments, then suddenly increases to around 45% for P_{H2}/P_{CO} = 3/1 to reach a maximum of around 65% for P_{H2}/P_{CO} = 0.5/1. When increasing the P_{H2}/P_{CO} ratio from low back to high, the oxygenates selectivity decreases, but remains above 30% for P_{H2}/P_{CO} between 30/1 and 5/1. Methane selectivity starts at above 75% for P_{H2}/P_{CO} = 30/1, then decreases rapidly with decreasing P_{H2}/P_{CO} ratio. Methane selectivity is below 15% when P_{H2}/P_{CO} is between 3/1 and 0.5/1. Counter-clockwise hysteresis is observed when increasing the P_{H2}/P_{CO} ratio from low back to high.

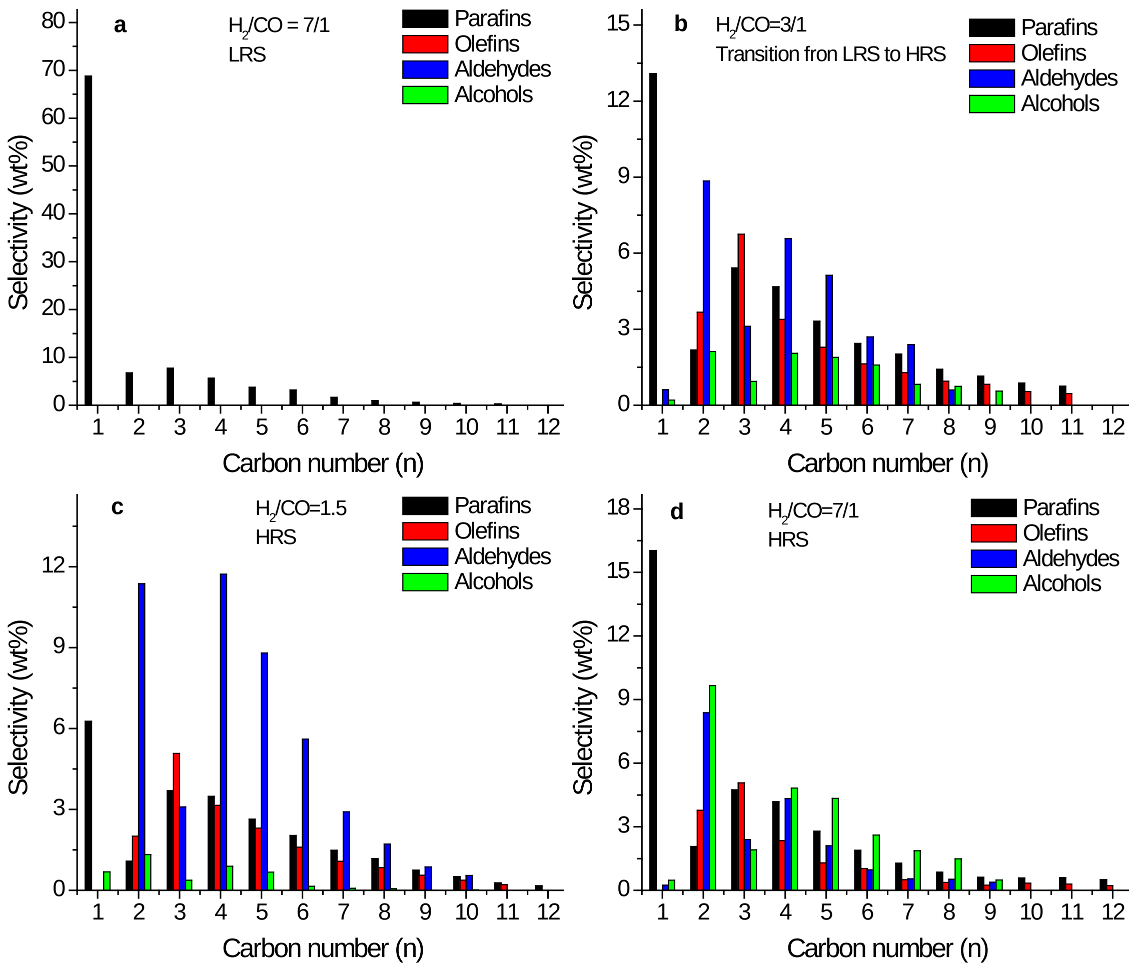

Supplementary Figure 3. Selected product distributions in CO hydrogenation on $Co_4Mn_1K_{0.1}$ catalyst at different reactivity states during kinetic hysteresis. (**a**) P_{H2}/P_{CO} = 7/1 at low reactivity state, (**b**) P_{H2}/P_{CO} = 3/1 at high reactivity state, (**c**) P_{H2}/P_{CO} = 1.5/1 at high reactivity state, and (**d**) P_{H2}/P_{CO} = 7/1 at high reactivity state. See also Figure 1 for detailed reaction conditions.

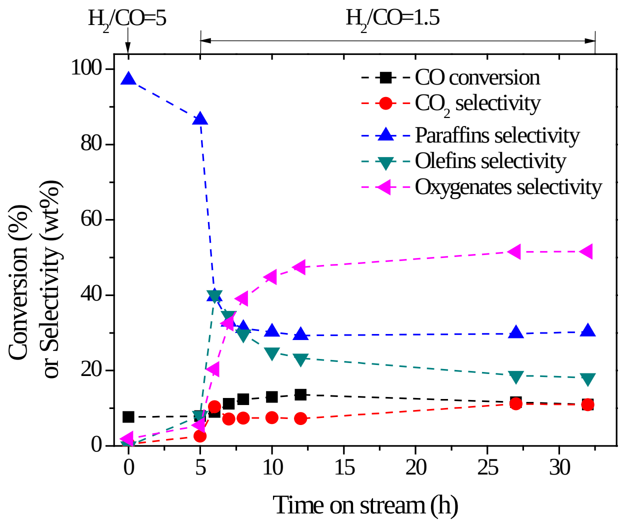

Supplementary Figure 4. Time dependent activity (CO conversion) and products selectivity after switching the P_{H2}/P_{CO} ratio from 5/1 to 1.5/1. The data clearly show a non-linear response behavior consisting in moderate changes of the products selectivity during the first 5 h time-on-stream (TOS) followed by a rapid drop of paraffins and raise of olefins (from < 5% to 40%) and oxygenates (from <5% to 20%) within a short period of time. For 6≤TOS≤12, oxygenates further increase to reach a selectivity of ~50% at the expense of both olefins and paraffins; close-to-steady state behavior for all product classes is obtained after ~ 12 h. Such selectivity response behavior is accompanied with a clear increase in the CO conversion (during 5≤TOS≤10) due to the restructuring of the catalyst causing a transition from the low reactivity state to the high reactivity state. Due to this restructuring, the CO conversion at P_{H2}/P_{CO} = 5 is lower than that at P_{H2}/P_{CO} = 1.5, as also shown in Figure 1a.

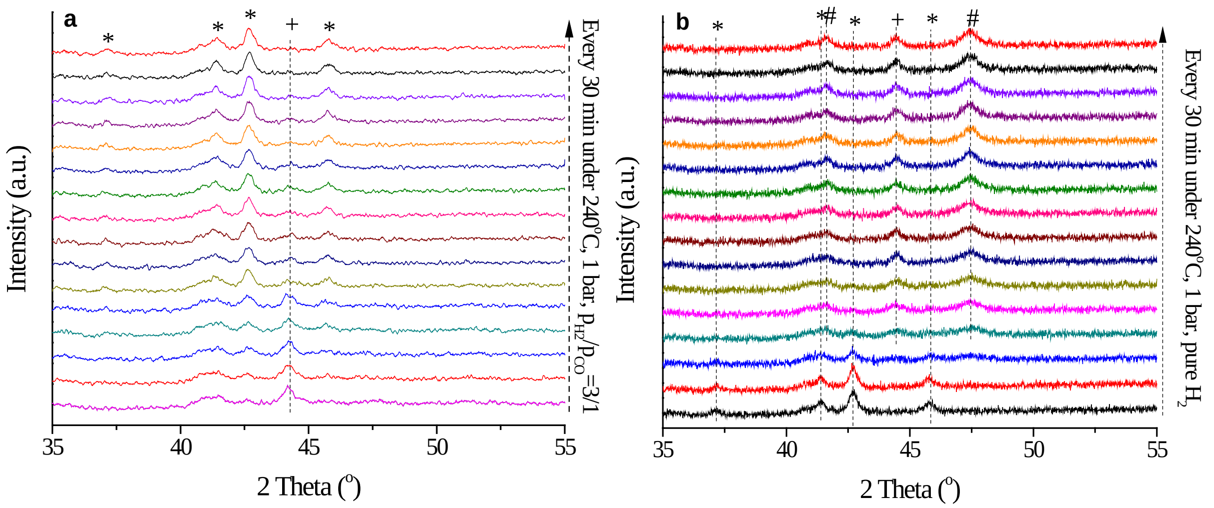

Supplementary Figure 5. Time-resolved (every 30 min) in-situ XRD patterns of the $Co_4Mn_1K_{0.1}$ catalyst. (**a**): dynamic process for the bulk Co_2C formation of the $Co_4Mn_1K_{0.1}$ sample when exposed to syngas at H_2/CO = 3/1, 240°C and atmospheric pressure. (**b**): dynamic fading for Co_2C when exposing the carbonized $Co_4Mn_1K_{0.1}$ sample to pure H_2 at the same temperature and pressure conditions. (*) Co_2C, (+) fcc Co, and (#) hcp Co. Starting with a mixed Co/Co_2C phase at P_{H2}/P_{CO}=3 (**a**), it is seen that while both, Co (fcc) and Co_2C are present initially (bottom line), the carbidic phase gains importance at the expense of the metallic one. Such chemical reconstructions of the catalyst bulk phase extend over several hours time-on-stream at P_{H2}/P_{CO}=3. The reversibility of the process is demonstrated by treating the catalyst in pure H_2 (**b**). The back-transformation of the carbidic phase becomes clearly visible after one hour time-on-stream. Both, Co hcp and fcc are formed this way.
