# Supplementary material for: Rate and selectivity hysteresis during the carbon monoxide hydrogenation over promoted Co/MnOx catalysts
Source: Nat Commun. 2019 Sep 2;10:3953. doi: 10.1038/s41467-019-11836-z (PMC6718517; doi:10.1038/s41467-019-11836-z)
Supplement: Supplementary file 2 — Peer Review File [file 41467_2019_11836_MOESM2_ESM.pdf]

Editorial Note: This manuscript has been previously reviewed at another journal that is not operating a transparent peer review scheme. This document only contains reviewer comments and rebuttal letters for versions considered at *Nature Communications*.

REVIEWERS' COMMENTS:

Reviewer #1 (Remarks to the Author):

This paper, possibly being transferred from Nature Catalysis, is acceptable with its present form, as my questions on the 1st and 2nd submitted manuscript to Nature Catalysis, are answered.

REVIEWERS' COMMENTS:

Reviewer #1 (Remarks to the Author):

This paper, possibly being transferred from Nature Catalysis, is acceptable with its present form, as my questions on the 1st and 2nd submitted manuscript to Nature Catalysis, are answered.

Reply: Thank you!
